# Supplementary figures and images for: Modulation of fracture healing by the transient accumulation of senescent cells
Source: eLife. 2021 Oct 7;10:e69958. doi: 10.7554/eLife.69958 (PMC8526061; doi:10.7554/eLife.69958)

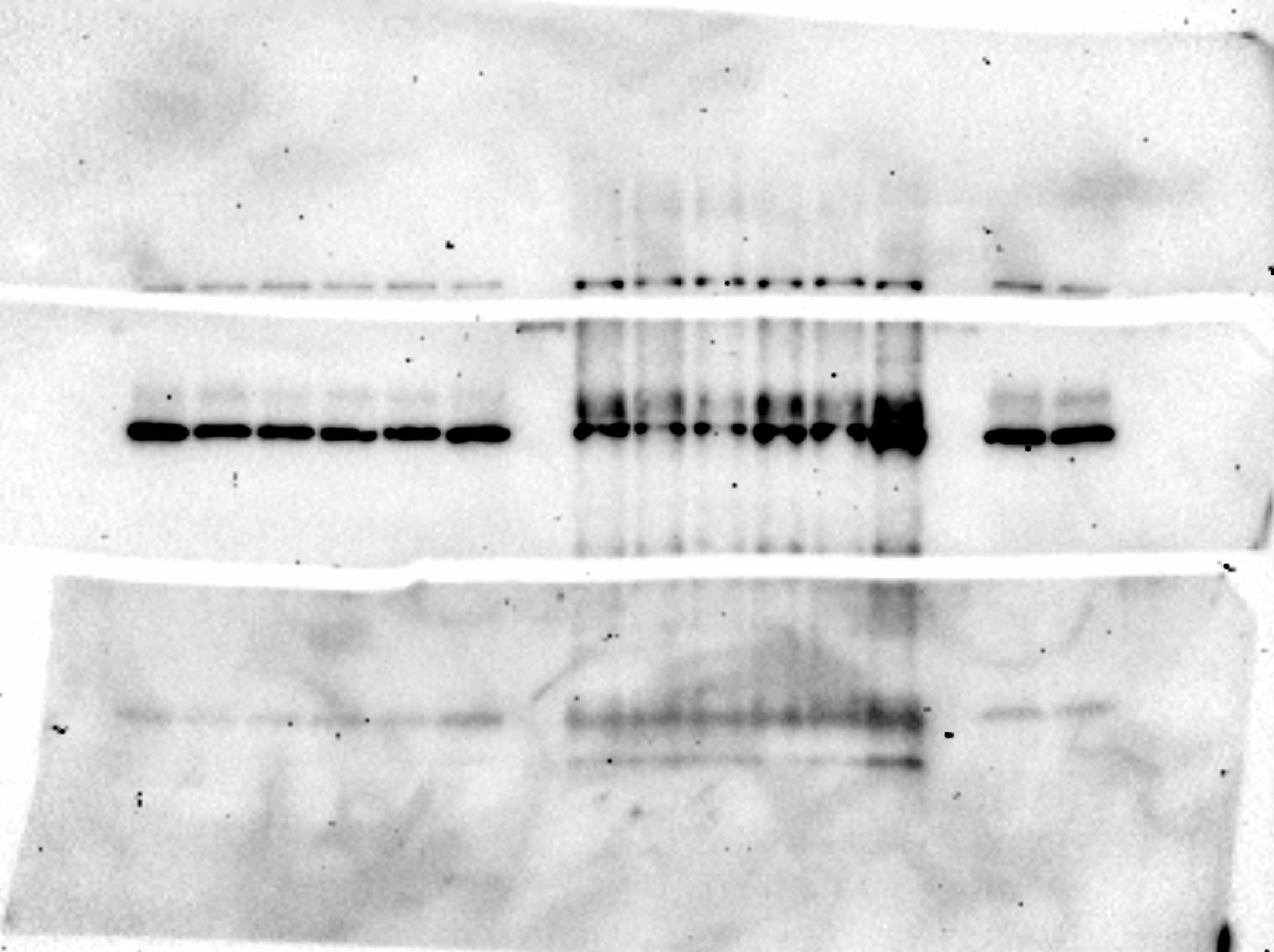

Supplement: Figure 6—source data 2. [file elife-69958-fig6-data2.zip › betaactin_western.tif]

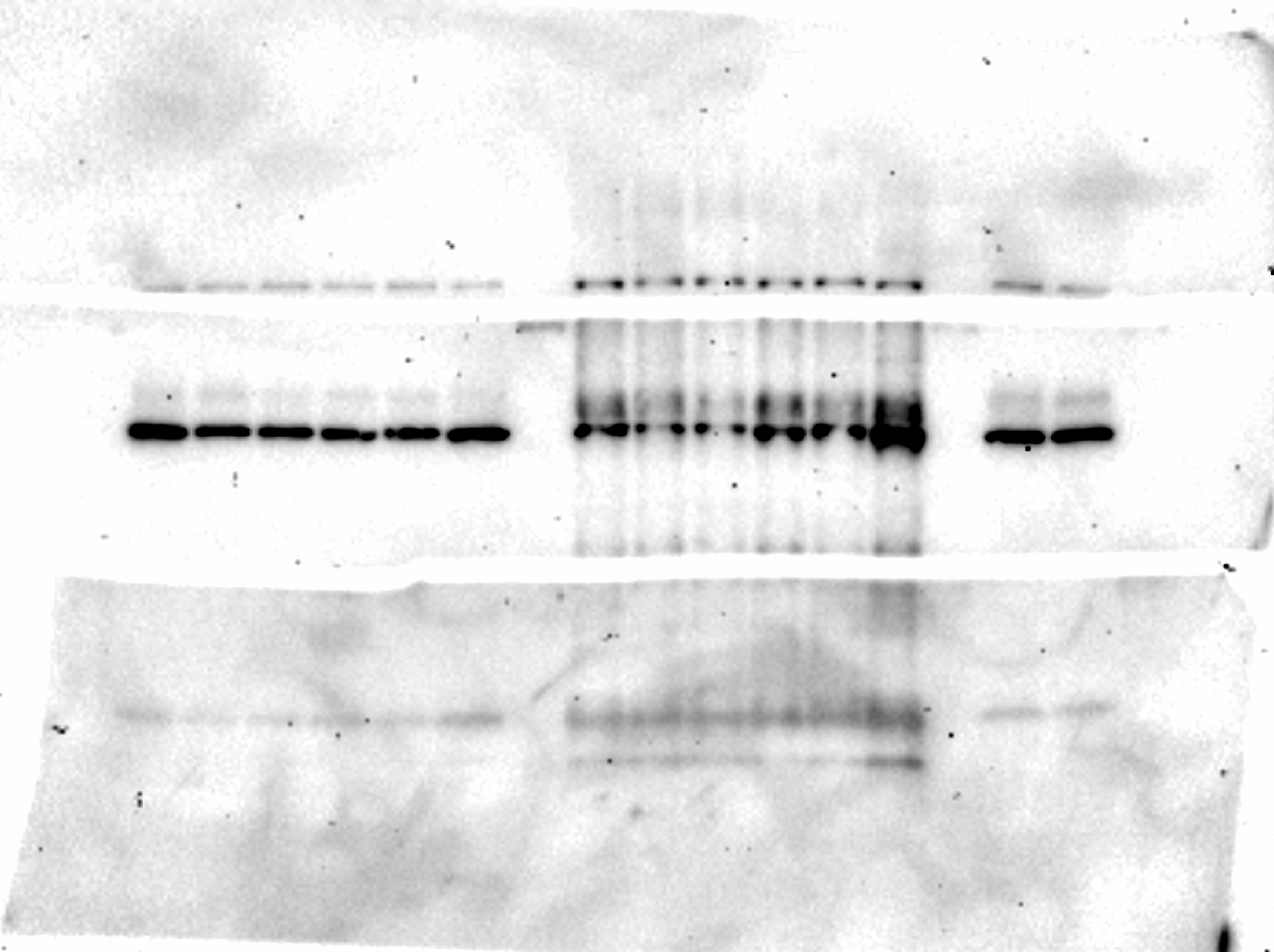

Supplement: Figure 6—source data 2. [file elife-69958-fig6-data2.zip › cdkn2a_western.tif]

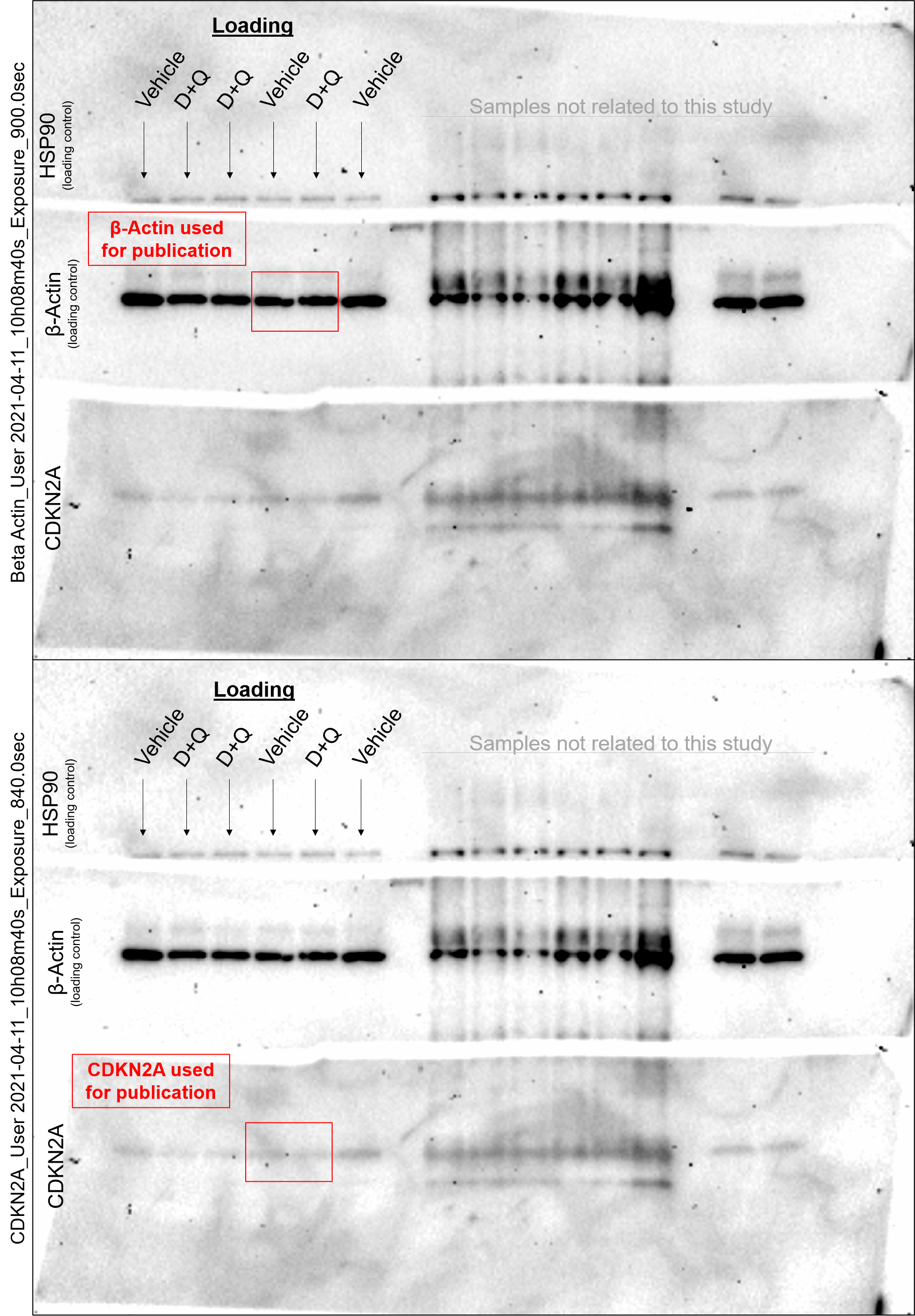

Supplement: Figure 6—source data 2. [file elife-69958-fig6-data2.zip › Figure6_panel_C_blots_raw.tif]
