# Supplementary material for: Modulation of fracture healing by the transient accumulation of senescent cells
Source: eLife. 2021 Oct 7;10:e69958. doi: 10.7554/eLife.69958 (PMC8526061; doi:10.7554/eLife.69958)
Supplement: Supplementary file 1. [file elife-69958-supp1.docx]

Supplementary File 1. Mouse primer sequences

| **Category** | **Gene** | **Forward Primer sequence (5’-3’)** | **Reverse Primer Sequence (5’-3’)** |
| --- | --- | --- | --- |
| Senescence | *Cdkn2a*^Ink4a^ | GAACTCTTTCGGTCGTACCC | AGTTCGAATCTGCACCGTAGT |
| Senescence | *Cdkn2a*^pan^ | AGCTCTTCTGCTCAACTACGG | GGAGAAGGTAGTGGGGTCCT |
| Senescence | *Cdkn1a*^Cip1^ | GAACATCTCAGGGCCGAAAA | TGCGCTTGGAGTGATAGAAATC |
| Senescence | *Tp53* | TCTTATCCGGGTGGAAGGAAA | GGCGAAAAGTCTGCCTGTCTT |
| SASP | *Actb* | AATCGTGCGTGACATCAAAGAG | GCCATCTCCTGCTCGAAGTC |
| SASP | *Ager* | CTGGCACTTAGATGGGAAACT | TGTCTCCTGGTCTCTTCCTT |
| SASP | *Ccl2* | GTCTGTGCTGACCCCAAGAAG | TGGTTCCGATCCAGGTTTTTA |
| SASP | *Ccl3* | TCCCAGCCAGGTGTCATTTT | TTGGAGTCAGCGCAGATCTG |
| SASP | *Ccl5* | GCCCACGTCAAGGAGTATTTCT | ACAAACACGACTGCAAGATTGG |
| SASP | *Ccl7* | CCCTGGGAAGCTGTTATCTTCA | CTGATGGGCTTCAGCACAGA |
| SASP | *Ccl8* | CCACACAGAAGTGGGTCAGTGA | TTCAAGGCTGCAGAATTTGAGA |
| SASP | *Csf1* | ATTGCCAAGGAGGTGTCAGAA | GGACCTTCAGGTGTCCATTCC |
| SASP | *Csf2* | CCTTGAACATGACAGCCAGCTA | CACAGTCCGTTTCCGGAGTT |
| SASP | *Csf3* | CCTGCAGGCTCTATCGGGTAT | ATCCAGCTGAAGCAAGTCCAA |
| SASP | *Cxcl1* | CCGAAGTCATAGCCACACTCAA | CAAGGGAGCTTCAGGGTCAAG |
| SASP | *Cxcl10* | TGAATCCGGAATCTAAGACCA | TTTTTGGCTAAACGCTTTCAT |
| SASP | *Cxcl12* | GCCAACGTCAAGCATCTGAAA | CAGCCGTGCAACAATCTGAA |
| SASP | *Cxcl15* | TCCATGGGTGAAGGCTACTGT | TTCATTGCCGGTGGAAATTC |
| SASP | *Cxcl16* | GCACCCCTGCACATAGTCAGA | AGGACAGTGCTCCTGATGGAA |
| SASP | *Cxcl2* | TCAAGGGCGGTCAAAAAGTT | CAGTTAGCCTTGCCTTTGTTCA |
| SASP | *Fas* | CTGCACCCTGACCCAGAATAC | ACAGCCAGGAGAATCGCAGTA |
| SASP | *Fasl* | CCGCTCTGATCTCTGGAGTGA | CACGAAGTACAACCCAGTTTCG |
| SASP | *Foxo4* | CCACGAAGCAGTTCAAATGCT | TTCAGACTCCGGCCTCATTG |
| SASP | *GAPDH* | GACCTGACCTGCCGTCTAGAAA | CCTGCTTCACCACCTTCTTGA |
| SASP | *Gas6* | AGGCTCAACTACACCCGAACA | TTTAACTTCCCAGGTGGTTTCC |
| SASP | *Gata4* | GCTCCTACTCCAGCCCCTAC | CAGGACTGGGCTGTCGAA |
| SASP | *Gdf15* | TGTGCAGGCAACTCTTGAAGA | GCGATACAGGTGGGGACACT |
| SASP | *Hmgb1* | TCCTTCGGCCTTCTTCTTGTT | AGGATGCTCGCCTTTGATTTT |
| SASP | *Icam1* | GTGGCGGGAAAGTTCCTGTT | GTCCAGCCGAGGACCATACA |
| SASP | *Ifng* | TTGGCTTTGCAGCTCTTCCT | ATGACTGTGCCGTGGCAGTA |
| SASP | *Igf1* | AAAAGCAGCCCGCTCTATCC | CTTCTGAGTCTTGGGCATGTCA |
| SASP | *Igfbp2* | GCCCCCTGGAACATCTCTACT | GTTGTACCGGCCATGCTTGT |
| SASP | *Igfbp3* | ACCTGCTCCAGGAAACATCAGT | TTTCCACACTCCCAGCATTG |
| SASP | *Igfbp4* | GCAACTTCCACCCCAAACAGT | CCTGTCTTCCGATCCACACA |
| SASP | *Il10* | TGGCTCAGCACTGCTATGCT | TGTACTGGCCCCTGCTGATC |
| SASP | *Il12a* | ATCCTGCTTCACGCCTTCAG | GATAGCCCATCACCCTGTTGA |
| SASP | *Il12b* | GCCAGTACACCTGCCACAAAG | TGTGGAGCAGCAGATGTGAGT |
| SASP | *Il15* | GGCATTCATGTCTTCATTTTGG | TCCAGTTGGCCTCTGTTTTAGG |
| SASP | *Il17a* | GGACTCTCCACCGCAATGAA | GCACTGAGCTTCCCAGATCAC |
| SASP | *Il1a* | AAGAGACCATCCAACCCAGATC | CCTGACGAGCTTCATCAGTTTG |
| SASP | *Il1b* | TCAGGCAGGCAGTATCACTCA | CACGGGAAAGACACAGGTAGCT |
| SASP | *Il3* | GCCTGCCTACATCTGCGAAT | CGAAAGTCATCCAGATCTCGAA |
| SASP | *Il4* | TCCTCACAGCAACGAAGAACAC | AAGCACCTTGGAAGCCCTACA |
| SASP | *Il6* | ACCACGGCCTTCCCTACTTC | TTGGGAGTGGTATCCTCTGTGA |
| SASP | *Il8* | TCCATGGGTGAAGGCTACTGT | TTCATTGCCGGTGGAAATTC |
| SASP | *Inhba* | CAGGAAGACACTGCACTTTGA | TTCAGGAAGAGCCACACTTCT |
| SASP | *Irf1* | CAGCCGAGACACTAAGAGCAAA | GAGAAAGTGTCCGGGCTAACAT |
| SASP | *Lif* | GCTGTATCGGATGGTCGCATA | TCTGGTCCCGGGTGATATTG |
| SASP | *Mif* | GCCACCATGCCTATGTTCATC | GGGTGAGCTCCGACAGAAAC |
| SASP | *Mmp12* | GTGCCCGATGTACAGCATCTT | GGTACCGCTTCATCCATCTTG |
| SASP | *Mmp13* | TGAGGAAGACCTTGTGTTTGCA | GCAAGAGTCGCAGGATGGTAGT |
| SASP | *Mmp2* | TGTGGGTGGAAATTCAGAAGGT | ACTTGTTGCCCAGGAAAGTGA |
| SASP | *Mmp7* | TTGCTGCCACCCATGAATTT | TCACAGTACCGGGAACAGAAGA |
| SASP | *Mmp8* | TGGCTGCTCATGAATTTGGA | CATCAAGGCACCAGGATCAGT |
| SASP | *Mmp9* | TGAGTCCGGCAGACAATCCT | CCCTGGATCTCAGCAATAGCA |
| SASP | *Nfkb1* | GGCTTTGCAAACCTGGGAAT | TCCGTGCTTCCAGTGTTTCA |
| SASP | *Pappa* | CATCTCAGGTGTGTCGAACCA | TGCAAGGATACCAAGCATGCT |
| SASP | *Pdgfa* | CTCGAAGTCAGATCCACAGCAT | CAGCCCCTACGGAGTCTATCTC |
| SASP | *Pdgfb* | GCTGAGCTGGACTTGAACATGA | CCTCGAGATGAGCTTTCCAACT |
| SASP | *Serpinb2* | TTCCGCATACTGGAAACATCAG | GGATGCGTCCTCAATCTCATC |
| SASP | *Serpine1* | GGACACCCTCAGCATGTTCA | CGGAGAGGTGCACATCTTTCT |
| SASP | *Sparc* | GAGGAGGTGGTGGCTGACAA | CACCTTGCCATGTTTGCAAT |
| SASP | *Spp1* | GAGGAGGTGGTGGCTGACAA | CACCTTGCCATGTTTGCAAT |
| SASP | *Tgfb1* | AGCGCTCACTGCTCTTGTGA | GCTGATCCCGTTGATTTCCA |
| SASP | *Tgfb2* | GGTGGCGCTCAGTCTGTCTAC | TCTTGCGCATAAACTGATCCAT |
| SASP | *Tgfb3* | CTGTCCACTTGCACCACGTT | CCTAATGGCTTCCACCCTCTT |
| SASP | *Tgfbr1* | CGTGTGCCAAATGAAGAGGAT | AAGGTGGTGCCCTCTGAAATG |
| SASP | *Tnf* | GTTCTGCAAAGGGAGAGTGG | GCACCTCAGGGAAGAGTCTG |
| SASP | *Tnfrsf11b* | CCAAGAGCCCAGTGTTTCTT | CCAAGCCAGCCATTGTTAAT |
| SASP | *Tnfrsf12a* | CCGCCGGAGAGAAAAGTTTAC | GGGTGCTCCTCACTGGATCA |
| SASP | *Tnfsf10* | CTCTCGGAAAGGGCATTCATT | TCGATGACCAGCTCTCCATTC |
| SASP | *Tnfsf11* | GCTGGGACCTGCAAATAAGT | TTGCACAGAAAACATTACACCTG |
| SASP | *Vcam1* | GGCTCCAGACATTTACCCAGTT | CATGAGCTGGTCACCCTTGAA |
| SASP | *Vegfa* | GTACCTCCACCATGCCAAGTG | TGGGACTTCTGCTCTCCTTCTG |
